# Long non-coding RNA EWSAT1 promotes human nasopharyngeal carcinoma cell growth in vitro by targeting miR-326/-330-5p

**DOI:** 10.18632/aging.101103

**Published:** 2016-11-03

**Authors:** Peng Song, Shu-Cheng Yin

**Affiliations:** ^1^ Department of Otorhinolaryngology-Head and Neck Surgery, ZhongNan Hospital of Wuhan University, Wuhan 430071, Hubei, P. R. China

**Keywords:** Ewing sarcoma associated transcript 1 (EWSAT1), hsa-miRNA-326/330-5p (miR-326/330-5p), cyclin D1, nasopharyngeal carcinoma (NPC), tumorigenesis

## Abstract

Long non-coding RNA (lncRNA) Ewing sarcoma associated transcript 1 (EWSAT1) has been identified as an oncogene, and its dysregulation is closed corrected with tumor progression in Ewing sarcoma. Recently, high-through put analysis reveals that EWSAT1 is also highly expressed in human nasopharyngeal carcinoma (NPC). However, whether the aberrant expression of EWSAT1 in NPC is corrected with malignancy or prognosis has not been expounded. Herein, we identified that EWSAT1 was up-regulated in NPC tissues and cell lines, and higher expression of EWSAT1 resulted in a markedly poorer survival time. EWSAT1 over-expression facilitated, while EWSAT1 silencing impaired cell growth in NPC. In addition, mechanistic analysis demonstrated that EWSAT1 up-regulated the expression of miR-326/330-5p clusters targeted gene cyclin D1 through acting as a competitive ‘sponge’ of miR-326/330-5p clusters. Collectively, our data revealed that EWSAT1 promotes NPC cell growth in vitro through up-regulating cyclin D1 partially via ‘spongeing’ miR-326/330-5p clusters.

## INTRODUCTION

Nasopharyngeal carcinoma (NPC), derived from the epithelial cells located in nasopharynx, displays a significant geographic distribution that with the highest incidence in Southern China and Southeast Asia [1,2]. Although the advance in radiotherapy techniques and chemotherapy regimens has markedly improved the local control of NPC [3], there are still some unknown reasons to hamper the treatment progress of NPC.

Recently, increasing evidence has revealed that long non-coding RNAs (lncRNAs) play crucial roles on several systems and might be critical to various types of known cancer genes [4–6]. A handle of studies [7–9] have reported that lncRNAs were crucial players in cancer biology, especially contributed to aberrant expression of gene products involved in the progress of numerous of human tumors [10–12]. Further, lncRNAs could be also regarded as prognostic or diagnostic markers considering their effects in clinical characteristics of tumor outcomes [8,11,13]. Never-theless, the clinical significance and biological mechanisms of lncRNAs in NPC progression are still remaining largely unknown.

Ewing sarcoma associated transcript 1 (EWSAT1, LINC00277, NR_026949), a kind of lncRNA located on chromosome 15 between 2 protein-coding genes, NOX5 and GLCE [7], is found up-expressed and functions an oncogenic role in Ewing sarcoma [7]. It has been reported that knockdown of EWSAT1 decreases soft agar colony growth of Ewing sarcoma cell lines [7], which indicated that EWSAT1 exerted an essential role on the occurrence, development and progression of malignant tumors. Recently, Yang and his colleagues has reported that EWSAT1 is highly expressed in NPC (6.85-fold than NP tissues) [14], while up to date, there is no related study elaborating the relevance between EWSAT1 expression and NPC progression. Hence, the role of EWSAT1 on NPC and its potential biological mechanisms still remain to be explored.

In our study, we are committed to explore the underlying molecular mechanism of EWSAT1 on NPC progression. We identified EWSAT1 harbors two conserved miR-326/330-5p clusters's cognate sites as the results of overlap for miRDB (http://mirdb.org/cgi-bin/custom_predict/customDetail.cgi) and PITA software (http://132.77.150.113/pubs/mir07/mir07_ prediction.html), and then we made an assumption that EWSAT1 might function as a competing endogenous RNA (ceRNA) for miR-326/330-5p clusters. Then, we searched microRNA.org, TargetScan, and PicTar for underlying targets of miR-326/330-5p that owned oncogenic characteristics, and found cyclin D1, a known oncogene, was an underlying target of miR-326/330-5p clusters. Collectively, we identified EWSAT1 might be a crucial oncogenic regulator involved in NPC progression via functioning as a ceRNA for miR-326/330-5p clusters, and in return initiating cyclin D1 pathway.

## RESULTS

### EWSAT1 is over-expressed and associated with prognosis in NPC

**Figure 1:**
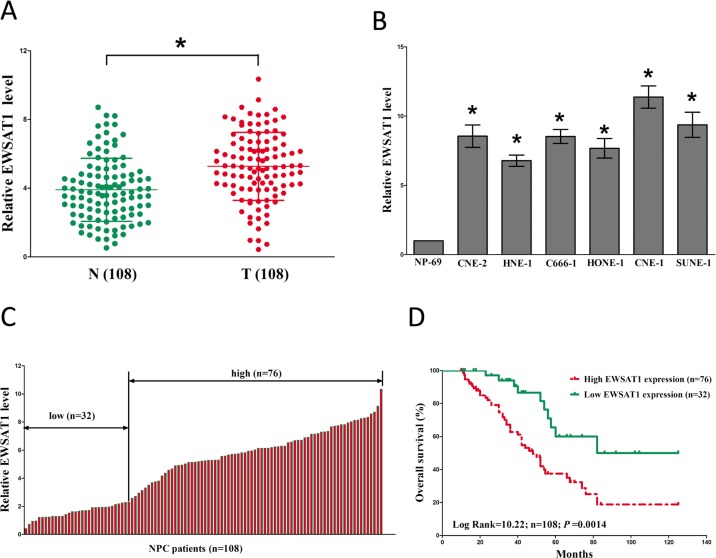
Relative EWSAT1 expression in NPC tissues and cell lines, and its clinical significance (**A**) Relative expression of EWSAT1 expression in NPC tissues (n = 108) and in paired adjacent normal tissues (n = 108). N represented Normal adjacent nasopharyngeal tissues, and T represented nasopharyngeal carcinoma tissues. EWSAT1 expression was examined by qPCR and normalized to GAPDH expression. (shown as ΔCT). (**B**) Relative expression of EWSAT1 expression in NPC cell lines and normal NP epidermal cell. (**C**) Relative expression of EWSAT1 expression in NPC tissues (n = 108) and in paired adjacent normal tissues (n = 108). EWSAT1 expression was examined by qPCR and normalized to GAPDH expression. (shown as ΔCT). (**D**) The Kaplan-Meier survival analysis indicated that EWSAT1 high expression (red line, n=76) has a worse overall survival compared to the low expression subgroup (green line, n=32). *P < 0.05. Means ± SEM are shown. Statistical analysis was conducted by student t-test.

**Figure 2:**
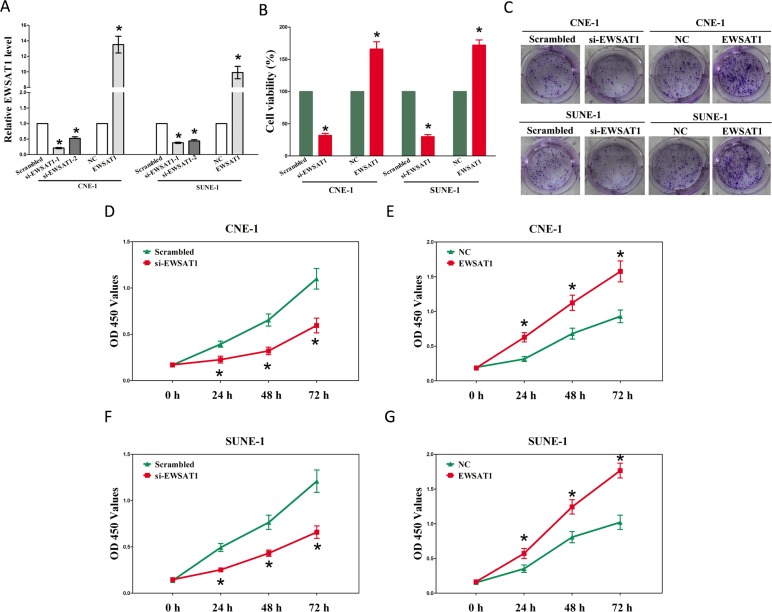
EWSAT1 promotes tumor NPC cell growth in vitro (**A-B**) Statistical analysis of trypan blue staining. (**C**) Shown is representative photomicrograph of colony formation assay after transfection for fourteen days. (**D-G**) CCK8 assays of CNE-1 and SUNE-1 cells after transfection. Assays were performed in triplicate. *P < 0.05. Means ± SEM are shown. Statistical analysis was conducted using student t-test.

To investigate the expression of EWSAT1 in NPC, qRT-PCR was conducted to examine EWSAT1 levels in human NPC tissues and their counterparts. Results revealed that EWSAT1 levels in 108 NPC tissues were significantly higher than that of in 108 counterparts (*P* <0.05) (Fig. 1A). Next, we examined EWSAT1 expression in NPC cell lines, and found that EWSAT1 was over-expressed in CNE-2, C666-1, HNE-1, CNE-1, SUNE-1, and HONE-1 cells, compared with that of in NP69 cells (a normal NP cell lines) (Fig. 1B). Among the six NPC cell lines, EWSAT1 are much higher expressed in CNE-1 and SUNE-1 cells, thus, CNE-1 and SUNE-1 cells were chose to conduct the following experiments. Then, NPC patients were divided into a high group (≥2.36-fold, n=76) and a low group (<2.36-fold, n=32) on the basis of the cutoff value of EWSAT1 expression (Fig. 1C). Moreover, Kaplan-Meier analysis indicated that high EWSAT1 expression was related to a poorer OS (log-rank test, *P* =0.0014, Fig. 2D). These results demonstrated that high EWSAT1 expression was related to poor prognosis, and over-expression of EWSAT1 might be essential in NPC progression.

### EWSAT1 promotes growth of NPC cells

Having known EWSAT1 is up-regulated and associated with poor prognosis in NPC. We then explore the oncogenic properties and roles of EWSAT1 on NPC. Firstly, we established NPC cell lines (CNE-1 and SUNE-1) with EWSAT1 stable over-expression or transient knockdown (Using RNAi). And then, trypan blue staining, colony formation as well as CCK8 assay were conducted to explore the role of EWSAT1 on NPC cell growth, and results demonstrated silence of EWSAT1 induced a reduction in the cell growth of CNE-1 and SUNE-1 cells than that of in their blank counterparts (Fig. 2A-C, D and F). However, overexpression of EWSAT1 exhibited a significant increase in the cell growth of CNE-1 and SUNE-1 cells than their blank counterparts (Fig. 2A-C, E and G). These results clearly demonstrate that EWSAT1 significantly facilitates cell growth in NPC cells.

### EWSAT1 functions as a ceRNA of miR-326/330-5p clusters in NPC

**Figure 3:**
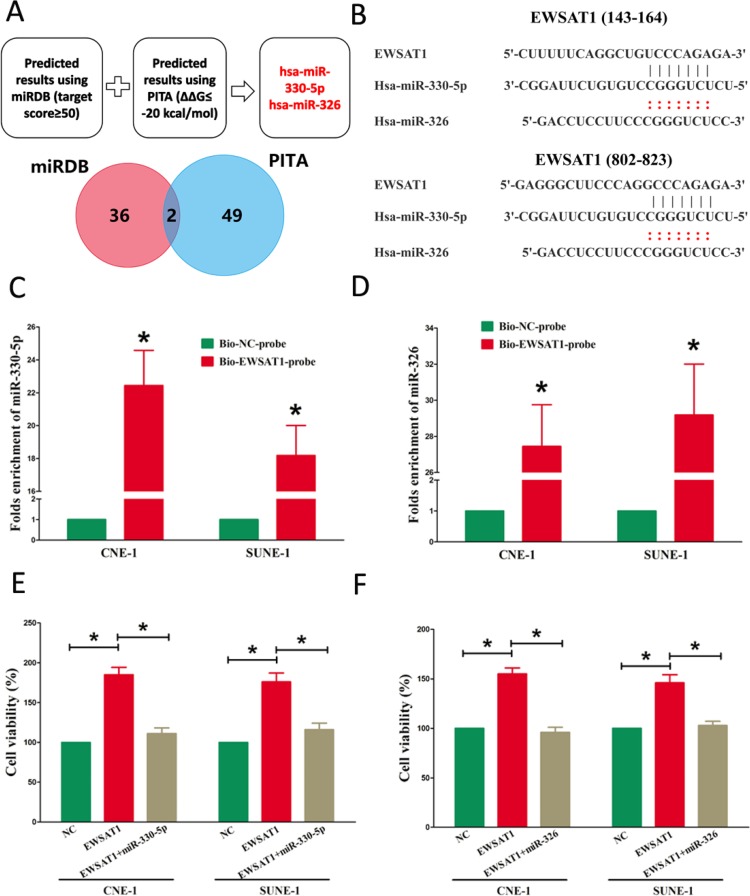
EWSAT1 is a direct target of miR-326/330-5p (**A**) Screen of the candidate miRNAs that target EWSAT1 predicted by miRDB and PITA. (**B**) Sequence alignment of miR-326/330-5p with the putative binding sites within the wild-type regions of EWSAT1. (**C-D**) Detection of miR-326/330-5p using qRT-PCR in the sample pulled down by biotinylated EWSAT1 probe. (**E-F**) Up-regulated miR-326/330-5p in CNE-1 and SUNE-1 cells, which stably over-expressed EWSAT1, largely reversed the favorable effects of EWSAT1 on cell proliferation. Assays were performed in triplicate. *P< 0.05. Means ± SEM are shown. Statistical analysis was conducted using student t-test.

**Figure 4:**
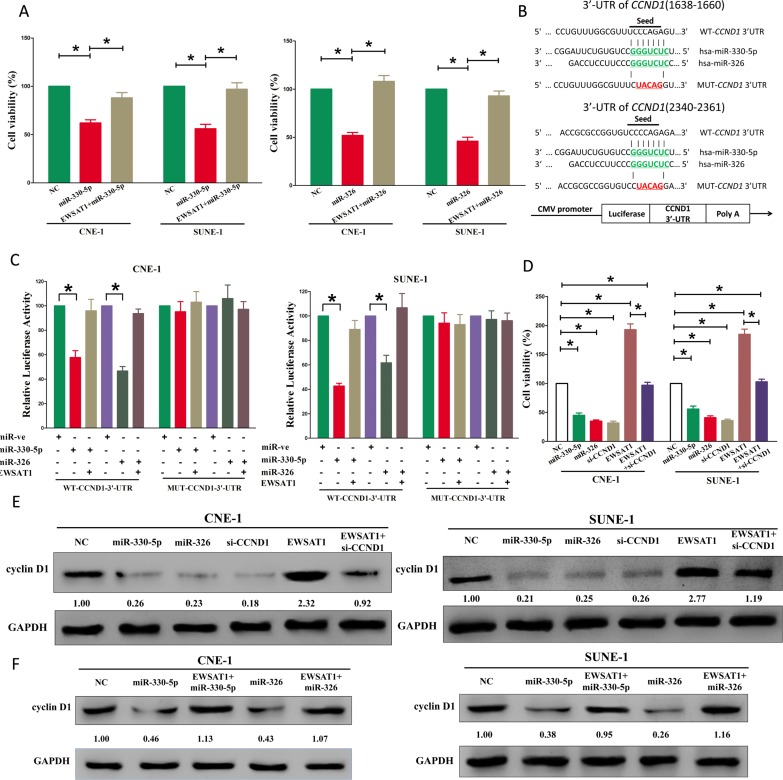
EWSAT1's oncogenic activity is in part through negative regulation of miR-326/330-5p, and then activation of cyclin D1 in NPC cells (**A**) Up-regulated EWSAT1 in miR-326/330-5p treated CNE-1 and SUNE-1 cells, significantly reversed the growth-inhibitory role of miR-326/330-5p in NPC cells. (**B**) The 3′-UTR of cyclin D1 harbors two miR-326/330-5p cognate sites. (**C**) Relative luciferase activity of reporter plasmids carrying wild-type or mutant cyclin D1 3′-UTR in CNE-1 and SUNE-1 cells co-transfected with negative control (NC) or miR-326/330-5p mimic. (D) Statistical analysis of trypan blue staining. (E-F) Protein expression of cyclin D1 in miR-330-5p, miR-330-5p+EWSAT1, miR-326, miR-326+ewsat1, si-CCND1, EWSAT1, or EWSAT1+si-CCND1 treated CNE-1 and SUNE-1 cells. Assays were performed in triplicate. *P < 0.05. Means ± SEM are shown. Statistical analysis was conducted using student One-Way ANOVA test.

Increasing of publications reported lncRNA might function as a ceRNA or a molecular sponge in regulating the biological functions of miRNA. To find miRNAs interacted with EWSAT1, we analyzed the overlap from results of miRDB (http://mirdb.org/cgi-bin/custom_predict/customDetail.cgi) and PITA software (http://132.77.150.113/pubs/mir07/mir07_ prediction.html) to predict potential miRNAs (results were shown in Table S1 and Table S2. In miRDB, miRNAs with target score≥50 were selected, and in PITA, miRNAs with target score target score ΔΔG≤-10 kcal/mol were selected, then intersection was conducted in the selected miRNAs in miRDB and PITA, and miR-326 and miR-330-5p were got as the candidate miRNAs (Table S1–2). To further verify whether miR-326/330-5p were enrichment in EWSAT1, we applied a pull-down assay by a biotin-labeled specific EWSAT1 probe. And biotin-NC probe was used as a negative control. qRT-PCR was conducted after precipitate. Results revealed that miR-326/330-5p were much richer in precipitate of EWSAT1 probe than that of in NC probe (Fig. 3C-D). These results reveal that miR-326/330-5p directly bind to EWSAT1 at the recognitive sites. Moreover, up-regulated miR-326/330-5p in CNE-1 and SUNE-1 cells, which stably over-expressed EWSAT1, significantly reversed the favorable roles of EWSAT1 on cell growth in NPC cells (Fig. 4A-B). These data indicated that EWSAT1 facilitated cell growth through binding miR-326/330-5p on NPC cells.

### EWSAT1's oncogenic roles are partially via spongeing miR-326/330-5p, and then activating cyclin D1

Having verified EWSAT1 was a target of miR-326/330-5p, the mechanism of miR-326/330-5p in EWSAT1-induced inhibition on NPC cells was still unknown. Both miR-326 and miR-330-5p repressed cell growth in NPC cell lines, while over-expressed EWSAT1 in miR-326 or miR-330-5p treated cells, significantly reversed the growth-inhibitory role of miR-326/330-5p in CNE-1 and SUNE-1 cells (Fig. 4A-B). These results confirmed that miR-326/330-5p made sense in EWSAT1-induced inhibitory roles on NPC cells. Having verified EWSAT1 could reversely regulate miR-326/330-5p expression, we then investigate its functional roles. To explore the function of miR-326/330-5p on NPC, we screen Targetscan, miRanda, PicTar to select potential predicted targets of miR-326/330-5p. We identified the top 100 potential targets, and among these genes, we found a well-known oncogene, cyclin D1, which was up-regulated in a large number of malignancies (Fig. 4B). These revealed that cyclin D1 could be a direct target of miR-326/330-5p in NPC. Next, we used luciferase reporter assays to verify whether cyclin D1 expression are really regulated by miR-326/330-5p, and results demonstrate that miR-326/330-5p inhibits luciferase activity in CNE-1 cells and SUNE-1 cells at the reporter plasmid with a WT cyclin D1 3′-UTR, but no significant inhibition was observed at the reporter plasmid with a mutant cyclin D1 3′-UTR (Fig. 4C). We next investigated the mechanism of miR-326/330-5p on NPC cell growth. Results of Trypan blue staining demonstrated both miR-326/330-5p and si-CCND1 treatment decreased cell growth of CNE-1 and SUNE-1 cells in comparison to that of in their blank counterparts (Fig. 4D), However, when treated CNE-1 and SUNE-1 cells with EWSAT1 plus si-CCND1, the favorable role of EWSAT1 on cell growth was inhibited by cyclin D1 knockdown, and the negative effect of si-CCND1 was alleviated by EWSAT1 over-expression (Fig. 4D). Additionally, we next investigated the effect of EWSAT1 and miR-326/330-5p on the protein expression of cyclin D1. Results revealed that both miR-326/330-5p and si-CCND1 treatment inhibited protein expression of cyclin D1, while EWSAT1 treatment significantly increased protein expression of cyclin D1 in CNE-1 and SUNE-1 cells (Fig. 4E-F), respectively. However, when treated CNE-1 and SUNE-1 cells with EWSAT1 plus si-CCND1, the favorable role of EWSAT1 on protein expression of cyclin D1 was inhibited by cyclin D1 knockdown, and the negative effect of si-CCND1 was alleviated by EWSAT1 over-expression (Fig. 4E-F). Our results reveal that miR-326/330-5p targets human cyclin D1 by directly binding to the predicted sites in 3′-UTR of cyclin D1 mRNA, and demonstrate that EWSAT1's oncogenic functions are partially through negative regulation of miR-326 /330-5p clusters, and then activation of cyclin D1.

## DISCUSSION

Recent studies have provided insights into the molecular mechanisms by which lncRNAs function in a variety of human tumors [36–38]. Marques *et al* reported EWSAT1-mediated gene repression facilitates Ewing sarcoma oncogenesis [7]. However, the roles and mechanisms of EWSAT1 in NPC have not been well elaborated. Yang and his colleagues have reported that EWSAT1 (LINC00277) is highly expressed in NPC (6.85-fold than NP tissues) [14]. Our present study added new evidence that over-expression of EWSAT1 owned oncogenic roles in NPC. EWSAT1 was revealed as a direct target of miR-326/330-5p clusters, and there was an interactive suppression between them. EWSAT1's function as an oncogene to facilitate tumor progression was partially attributed to its ability to acting as a ceRNA for miR-326/330-5p clusters, and subsequent to activating of the cyclin D1 signaling pathway in NPC. Thus, our study contributes to an increasing of literatures supporting the importance of non-annotated lncRNA species in the field of cancer research.

This report is the first time to directly explore the association between EWSAT1 expression and NPC. Herein, we found EWSAT1 expression in NPC tissues was significantly higher than that of in NP tissues. Our study also revealed a correction between EWSAT1 levels and NPC prognosis or therapeutic outcome. A strong correction of high EWSAT1 expression in tumors with poor survival was confirmed in 108 NPC samples, revealing that EWSAT1 expression levels could be as a useful prognostic biomarker to help identify patients who are at a higher risk of NPC progression. In addition, EWSAT1 overexpression significantly increased NPC cell viability and growth in vitro, while EWSAT1 knockdown reversed it. In conclusion, our data indicate that EWSAT1 may function as an oncogene and play a critical effect in NPC development and progression.

Although EWSAT1 has been suggested to act as an oncogene, the underlying mechanism by which EWSAT1-mediated gene expression participates in tumorigenesis remains to be clarified. Marques *et al* reported EWSAT1 controlled gene expression partially through an interaction with HNRNPK, and functioned as an oncogene through regulating gene expression downstream of EWS-FLI1 in Ewing sarcoma [7]. In our present study, we aimed to discover another underlying molecular mechanism of EWSAT1 on NPC progression, namely, functioning as “molecular sponges” to regulate microRNAs. It was reported that LncRNAs played a crucial effect in multiple processes in cells through acting as ceRNAs to regulate microRNAs [39]. A handle of lncRNAs have been evaluated, including GAS5 [40,41], NEAT1 [8], and CCAT1 [42] and so on. In our study, we investigated the effect of EWSAT1 in NPC cell lines and discovered that EWSAT1 involved in the ceRNA regulatory network and functioned as endogenous miRNA sponges to bind to miR-326/330-5p and regulated its function. Recent studies indicated miR-326/330-5p showed tumor suppressive role on lung cancer [43–46], breast cancer [47], malignant melanoma [48], colorectal cancer [49], and glioblastoma [50,51], while their role on NPC had not been investigated. In our present study, miR-326/330-5p inhibited growth in NPC cell lines. More-over, biotin-avidin pull-down system demonstrated EWSAT1 could pull down miR-326/330-5p. In addition, our study also revealed that miR-326/330-5p could reverse the favorable roles of EWSAT1 on cell growth in NPC cell lines, which demonstrated EWSAT1 played its favorable role on NPC progression, at least in part, through inhibiting miR-326/330-5p clusters.

Having shown the critical role of miR-326/330-5p on suppressing NPC progression, we searched for the potential gene effectors involved in its function. MiR-326/330-5p can regulate numerous of target genes. Recent study indicated that miR-330-5p inhibits proliferation and migration of keratinocytes by targeting Pdia3 expression [52], and regulates tyrosinase and PDIA3 expression and suppresses cell proliferation and invasion in cutaneous malignant melanoma [48]. MiR-326 reverses chemoresistance in human lung adeno-carcinoma cells by targeting specificity protein 1 [53], and regulates cell proliferation and migration in lung cancer by targeting phox2a and is regulated by HOTAIR [54]. But among all of the predicted target genes for miR-326/330-5p clusters, we found that cyclin D1 acted as a crucial effector of miR-326/330-5p. Aberrant cyclin D1 expression has been associated to several types of cancers [44,55–62]. Using bio-informatics, we verified cyclin D1 as a direct target of miR-326/330-5p, and luciferase reporter assays confirmed that miR-326/330-5p targeted cyclin D1 mRNA at its 3′-UTR. Moreover, our results also demonstrated miR-326/330-5p clusters exerted its tumor suppressive role on NPC through targeting cyclin D1. Interesting, we found that when treated CNE-1 and SUNE-1 cells with EWSAT1 plus si-CCND1, the negative effect of si-CCND1 was alleviated by EWSAT1 over-expression. In addition, EWSAT1 facilitated NPC cell growth through up-regulated the expression of cyclin D1. Those effects could be partially attributed to EWSAT1 functioning as a ceRNA for miR-326/330-3p. Another possible mechanism for them may be due to the possibility that EWSAT1 increased CCND1 mRNA through directly or indirectly binding at the CCND1 promoter, and then activating its transcription.

In conclusion, our data reveal that high-expressed EWSAT1 is an oncogenic lncRNA that facilitates the oncogenisis and progression of NPC through miR-326/330-5p-cyclin D1 axis. EWSAT1 may also function as a prognostic factor in NPC. The present results elucidate an underlying mechanism of the oncogenic role for EWSAT1 in NPC, and imply that EWSAT1 could be used as a marker and potential therapeutic target in NPC.

## MATERIALS AND METHODS

### Ethics statement

For the analyzed tissue specimens, all patients gave informed consent to use excess pathological specimens for research purposes. The protocols employed in this Subjects Committee. The use of human tissues was approved by the institutional review board of the Wuhan University and conformed to the Helsinki Declaration and to the local legislation. Patients offering samples for the study signed informed consent forms.

### Tissue collection

108 cases of fresh NPC tissues and 108 non-cancerous nasopharyngitis (NP) tissues were snap-frozen and stored in liquid nitrogen until further use for qRT-PCR assay. Elective surgery was carried out on these patients at ZhongNan Hospital of Wuhan University (Wuhan, China). The use of tissues for this study has been approved by the ethics committee of ZhongNan Hospital of Wuhan University. Before using these clinical materials for research purposes, all the patients signed the informed consent. None of these patients received any pre-operative chemotherapy or radio-therapy.

### Cell culture and transfection

The human NPC cell lines, namely, SUNE-1, CNE-1, HNE-1, CNE-2, C666-1 and HONE-1 were all purchased from Cell bank of Chinese academy of sciences, and were cultured in RPMI-1640 (Invitrogen, Carlsbad, CA, USA) supplemented with 10%. fetal bovine serum (FBS). The human immortalized nasopharyngeal epithelial cell line NP69 (from Cell bank of Chinese academy of sciences) was cultured in keratinocyte/serum-free medium (Invitrogen) supplemented with bovine pituitary extract. pcDNA3.1-CT-GFP-EWSAT1 (NR_026949), si-EWSAT1, miR-326 (MIMAT0000756), miR-330-5p (MIMAT0000751), or si-CCND1 (NM_053056), were purchased from GenePharma Co., Ltd. (Shanghai, China). Complete medium without antibiotics was used to culture the cells at least twenty-four hours prior to transfection. The cells were washed with 1× PBS (pH7.4) and then transiently transfected with 100 nM NC or pcDNA3.1-CT-GFP-EWSAT1, si-EWSAT1, miR-326, miR-330-5p, or si-CCND1, using Lipofectamine™ 2000 (Invitrogen, Carlsbad, CA, USA) according to the manufacturer's instructions.

### Cell transfection and stable cell lines

Cells were transfected with DNA plasmids using transfast transfection reagent lipofectamineR 2000 (Invitrogen) according to manufacturer's instructions [15–17]. For screening stable cell lines, forty-eight hours after transfection, cells were plated in the selective medium containing G418 (1000–2000 μg/ml, Invitrogen, Ltd., U.K) for the next 4 weeks or so, and the selective media were replaced every 3 days.

### Western blot analysis

Total proteins were extracted from cultured cells using RIPA buffer containing PMSF and quantified using BCA protein assay kit (Beyotime, Haimen, China)[18–22]. Protein lysates were subjected to SDS-PAGE and transferred onto polyvinylidenedifluoride (PVDF) membrane (Millipore, Billerica, MA) followed by incubating with a primary antibody, and then a secondary antibody. The signals were detected with KeyGEN Enhanced ECL detection kit according to the manufacturer's instructions (KeyGEN, NanJing, China). Rabbit polyclonal antibody against human cyclin D1 and GAPDH were purchased from Abcam.

### qRT-PCR

Total RNA was extracted with TRIzol reagent in accordance with the manufacturer's instructions (Invitrogen, CA, USA)[23–27]. cDNA was synthesised with the PrimeScript RT reagent Kit (Promega, Madison, WI, USA). Real-time PCR was carried out in a total volume of 10 μl, including 8 μl of TaqMan Power SYBR Green PCR Mix (Invitrogen), 0.5 μl of each primer at 25 μM, and 1 μl of cDNA. The quantitative RT-PCR was carried out on the Roche LightCycler® 96 (LC96) real-time PCR platform using the 2^−ΔΔCT^ method. Gene expression results were normalized by internal control GAPDH. Each sample was tested in triplicate.

### Colony formation assay

CNE-1 and SUNE-1 cells and their control cells were placed in six-well plate (300 cells/well) and cultured for 2 weeks. Colonies were fixed with methanol and stained with 0.1% crystal violet in 20% methanol for 15 min. Colonies larger than 0.1 mm diameter were scored. The experiment was performed in triplicate for each cell line.

### Luciferase reporter assays

Oligonucleotides containing the wild-type (WT) or mutant (MT) puptative miR-330-5p or miR-326 binding sites of the 3′-untranslated regions (3′-UTR) of the CCND1 mRNA were ligated into the pMIR-REPORT luciferase reporter plasmid vector (see in http://www.addgene.org/browse/sequence_vdb/3582/), respectively. Restriction enzymes NotI and XhoI were adopted and the sticky ends were GCGGCCGC, CTCGAG. The primers for 3′-UTR CCND1 (1620-2390) were: F: 5′-AATGCGGCCGCCACAAAGACATTGATTCAGC-3′; R: 5′-GGCGGCTCGAGGCAGGGAAGAGAAGAGGGAC-3′. 4×10^4^ cells per well were seeded in 24-well plates in triplicate. 24 h later, 100 ng firefly luciferase construct was co-transfected with 10 ng pRL-TK renilla plasmid into cells using Lipofectamine 2000 reagent (Invitrogen) in the presence of microRNA mimics or corresponding negative control. Media were replaced at 6 h, and the luciferase and renilla signals were measured 48 h after transfection using the Dual Luciferase Reporter Assay Kit (Promega), according to the manufacturer's protocol. The experiments were performed independently in triplicate.

### CCK8 assay

CCK8 Assay was carried out using the protocol described previously [28–35]. Briefly, cell growth was measured using the cell proliferation reagent WST-8 (Roche Biochemicals, Mannheim, Germany). After plating cells in 96-well microtiter plates (Corning Costar, Corning, NY) at 1.0× 10 ^3^ /well, 10 μL of CCK8 was added to each well at the time of harvest, according to the manufacturer's instructions. One hour after adding CCK8, cellular viability was determined by measuring the absorbance of the converted dye at 450 nm.

### Trypan blue staining

Cell viability was assessed using the trypan blue (Lonza, Basel, Switzerland) exclusion method. The CNE-1 and SUNE-1 cells were seeded in 24-well culture plates at a density of 3 × 10^5^ cells per well, and the cells were then transfected with Vector, miR-326, miR-330-5p, pcDNA3.1-CT-GFP-EWSAT1, miR-326 plus pcDNA3.1-CT-GFP-EWSAT1, or miR-330-5p plus pcDNA3.1-CT-GFP-EWSAT1. Each cell sus-pension was mixed with an equal volume of 0.4% trypan blue solution, and the living cells were quantified using a hemocytometer. The cells were also counted using a microscope. The data are representative of three independent experiments performed on different days.

### RNA pull-down assays

EWSAT1 transcripts were transcribed using T7 RNA polymerase (Ambio life) *in vitro*, then by using the RNeasy Plus Mini Kit (Qiagen) and treated with DNase I (Qiagen). Purified RNAs were biotin-labeled with the Biotin RNA Labeling Mix (Ambio life). Positive control, negative control and Biotinylated RNAs were mixed and incubated with CNE-1 and SUNE-1 cell lysates. Then, magnetic beads were added to each binding reaction, and incubated at room temperature. Finally, the beads were washed, and the eluted proteins were detected by western blot analysis.

### Statistical analysis

All experiments were repeated for three times independently. Results were shown as the means ± standard error mean (SEM). Two independent sample t-test or One-Way Analysis of Variance (ANOVA) was performed using SPSS 20.0 software to assess significant differences in measured variables among groups. A value of *P* <0.05 was considered to indicate a statistically significant difference.

## SUPPLEMENTARY MATERIAL TABLES





## References

[1] Wei KR, Zheng RS, Zhang SW, Liang ZH, Ou ZX, Chen WQ (2014). Nasopharyngeal carcinoma incidence and mortality in China in 2010. Chin J Cancer.

[2] Chen YP, Wang ZX, Chen L, Liu X, Tang LL, Mao YP, Li WF, Lin AH, Sun Y, Ma J (2015). A Bayesian network meta-analysis comparing concurrent chemoradiotherapy followed by adjuvant chemotherapy, concurrent chemoradiotherapy alone and radiotherapy alone in patients with locoregionally advanced nasopharyn-geal carcinoma. Ann Oncol.

[3] Chen YP, Wang ZX, Chen L, Liu X, Tang LL, Mao YP, Li WF, Lin AH, Sun Y, Ma J (2015). A Bayesian network meta-analysis comparing concurrent chemoradiotherapy followed by adjuvant chemotherapy, concurrent chemoradiotherapy alone and radiotherapy alone in patients with locoregionally advanced nasopharyn-geal carcinoma. Ann Oncol.

[4] Ørom UA, Derrien T, Beringer M, Gumireddy K, Gardini A, Bussotti G, Lai F, Zytnicki M, Notredame C, Huang Q, Guigo R, Shiekhattar R (2010). Long noncoding RNAs with enhancer-like function in human cells. Cell.

[5] Geisler S, Lojek L, Khalil AM, Baker KE, Coller J (2012). Decapping of long noncoding RNAs regulates inducible genes. Mol Cell.

[6] Lin A, Li C, Xing Z, Hu Q, Liang K, Han L, Wang C, Hawke DH, Wang S, Zhang Y, Wei Y, Ma G, Park PK (2016). The LINK-A lncRNA activates normoxic HIF1α signalling in triple-negative breast cancer. Nat Cell Biol.

[7] Marques Howarth M, Simpson D, Ngok SP, Nieves B, Chen R, Siprashvili Z, Vaka D, Breese MR, Crompton BD, Alexe G, Hawkins DS, Jacobson D, Brunner AL (2014). Long noncoding RNA EWSAT1-mediated gene repression facilitates Ewing sarcoma oncogenesis. J Clin Invest.

[8] Sun C, Li S, Zhang F, Xi Y, Wang L, Bi Y, Li D (2016). Long non-coding RNA NEAT1 promotes non-small cell lung cancer progression through regulation of miR-377-3p-E2F3 pathway. Oncotarget.

[9] Zang C, Nie FQ, Wang Q, Sun M, Li W, He J, Zhang M, Lu KH (2016). Long non-coding RNA LINC01133 represses KLF2, P21 and E-cadherin transcription through binding with EZH2, LSD1 in non small cell lung cancer. Oncotarget.

[10] Khorkova O, Hsiao J, Wahlestedt C (2015). Basic biology and therapeutic implications of lncRNA. Adv Drug Deliv Rev.

[11] Yuan SX, Wang J, Yang F, Tao QF, Zhang J, Wang LL, Yang Y, Liu H, Wang ZG, Xu QG, Fan J, Liu L, Sun SH, Zhou WP (2016). Long noncoding RNA DANCR increases stemness features of hepatocellular carcinoma by derepression of CTNNB1. Hepatology.

[12] Wang F, Yuan JH, Wang SB, Yang F, Yuan SX, Ye C, Yang N, Zhou WP, Li WL, Li W, Sun SH (2014). Oncofetal long noncoding RNA PVT1 promotes proliferation and stem cell-like property of hepatocellular carcinoma cells by stabilizing NOP2. Hepatology.

[13] Lu Z, Xiao Z, Liu F, Cui M, Li W, Yang Z, Li J, Ye L, Zhang X (2016). Long non-coding RNA HULC promotes tumor angiogenesis in liver cancer by up-regulating sphingosine kinase 1 (SPHK1). Oncotarget.

[14] Yang QQ, Deng YF (2015). Genome-wide analysis of long non-coding RNA in primary nasopharyngeal carcinoma by microarray. Histopathology.

[15] Hiraki M, Nishimura J, Takahashi H, Wu X, Takahashi Y, Miyo M, Nishida N, Uemura M, Hata T, Takemasa I, Mizushima T, Soh JW, Doki Y (2015). Concurrent Targeting of KRAS and AKT by MiR-4689 Is a Novel Treatment Against Mutant KRAS Colorectal Cancer. Mol Ther Nucleic Acids.

[16] Stiuso P, Potenza N, Lombardi A, Ferrandino I, Monaco A, Zappavigna S, Vanacore D, Mosca N, Castiello F, Porto S, Addeo R, Prete SD, De Vita F (2015). MicroRNA-423-5p Promotes Autophagy in Cancer Cells and Is Increased in Serum From Hepato-carcinoma Patients Treated With Sorafenib. Mol Ther Nucleic Acids.

[17] Misso G, Di Martino MT, De Rosa G, Farooqi AA, Lombardi A, Campani V, Zarone MR, Gullà A, Tagliaferri P, Tassone P, Caraglia M (2014). Mir-34: a new weapon against cancer?. Mol Ther Nucleic Acids.

[18] Yang C, Sun C, Liang X, Xie S, Huang J, Li D (2016). Integrative analysis of microRNA and mRNA expression profiles in non-small-cell lung cancer. Cancer Gene Ther.

[19] Sun C, Yang C, Xue R, Li S, Zhang T, Pan L, Ma X, Wang L, Li D (2015). Sulforaphane alleviates muscular dystrophy in mdx mice by activation of Nrf2. J Appl Physiol (1985).

[20] Sun CC, Li SJ, Yang CL, Xue RL, Xi YY, Wang L, Zhao QL, Li DJ (2015). Sulforaphane Attenuates Muscle Inflammation in Dystrophin-deficient mdx Mice via NF-E2-related Factor 2 (Nrf2)-mediated Inhibition of NF-κB Signaling Pathway. J Biol Chem.

[21] Sun C, Li S, Li D (2016). Sulforaphane mitigates muscle fibrosis in mdx mice via Nrf2-mediated inhibition of TGF-β/Smad signaling. J Appl Physiol (1985).

[22] Fang J, Sun CC, Gong C (2016). Long noncoding RNA XIST acts as an oncogene in non-small cell lung cancer by epigenetically repressing KLF2 expression. Biochem Biophys Res Commun.

[23] Zeng Z, Tung CH, Zu Y (2014). A cancer cell-activatable aptamer-reporter system for one-step assay of circulating tumor cells. Mol Ther Nucleic Acids.

[24] Sun H, Zhu X, Lu PY, Rosato RR, Tan W, Zu Y (2014). Oligonucleotide aptamers: new tools for targeted cancer therapy. Mol Ther Nucleic Acids.

[25] Arif T, Vasilkovsky L, Refaely Y, Konson A, Shoshan-Barmatz V (2014). Silencing VDAC1 Expression by siRNA Inhibits Cancer Cell Proliferation and Tumor Growth In Vivo. Mol Ther Nucleic Acids.

[26] Arif T, Vasilkovsky L, Refaely Y, Konson A, Shoshan-Barmatz V (2014). Silencing VDAC1 Expression by siRNA Inhibits Cancer Cell Proliferation and Tumor Growth In Vivo. Mol Ther Nucleic Acids.

[27] Chi Y, Wang X, Yang Y, Zhang C, Ertl HC, Zhou D (2014). Survivin-targeting Artificial MicroRNAs Mediated by Adenovirus Suppress Tumor Activity in Cancer Cells and Xenograft Models. Mol Ther Nucleic Acids.

[28] Sun C, Liu Z, Li S, Yang C, Xue R, Xi Y, Wang L, Wang S, He Q, Huang J, Xie S, Jiang W, Li D (2015). Down-regulation of c-Met and Bcl2 by microRNA-206, activates apoptosis, and inhibits tumor cell proliferation, migration and colony formation. Oncotarget.

[29] Sun C, Sang M, Li S, Sun X, Yang C, Xi Y, Wang L, Zhang F, Bi Y, Fu Y, Li D (2015). Hsa-miR-139-5p inhibits proliferation and causes apoptosis associated with down-regulation of c-Met. Oncotarget.

[30] Sun C, Liu Z, Li S, Yang C, Xue R, Xi Y, Wang L, Wang S, He Q, Huang J, Xie S, Jiang W, Li D (2015). Down-regulation of c-Met and Bcl2 by microRNA-206, activates apoptosis, and inhibits tumor cell proliferation, migration and colony formation. Oncotarget.

[31] Sun CC, Li SJ, Li DJ (2016). Hsa-miR-134 suppresses non-small cell lung cancer (NSCLC) development through down-regulation of CCND1. Oncotarget.

[32] Sun C, Sang M, Li S, Sun X, Yang C, Xi Y, Wang L, Zhang F, Bi Y, Fu Y, Li D (2015). Hsa-miR-139-5p inhibits proliferation and causes apoptosis associated with down-regulation of c-Met. Oncotarget.

[33] Sun C, Huang C, Li S, Yang C, Xi Y, Wang L, Zhang F, Fu Y, Li D (2016). Hsa-miR-326 targets CCND1 and inhibits non-small cell lung cancer development. Oncotarget.

[34] Sun CC, Li SJ, Zhang F, Pan JY, Wang L, Yang CL, Xi YY, Li J (2016). Hsa-miR-329 exerts tumor suppressor function through down-regulation of MET in non-small cell lung cancer. Oncotarget.

[35] Sun C, Li S, Yang C, Xi Y, Wang L, Zhang F, Li D (2016). MicroRNA-187-3p mitigates non-small cell lung cancer (NSCLC) development through down-regulation of BCL6. Biochem Biophys Res Commun.

[36] Barnhill LM, Williams RT, Cohen O, Kim Y, Batova A, Mielke JA, Messer K, Pu M, Bao L, Yu AL, Diccianni MB (2014). High expression of CAI2, a 9p21-embedded long noncoding RNA, contributes to advanced-stage neuroblastoma. Cancer Res.

[37] Nie FQ, Zhu Q, Xu TP, Zou YF, Xie M, Sun M, Xia R, Lu KH (2014). Long non-coding RNA MVIH indicates a poor prognosis for non-small cell lung cancer and promotes cell proliferation and invasion. Tumour Biol.

[38] Yang F, Huo XS, Yuan SX, Zhang L, Zhou WP, Wang F, Sun SH (2013). Repression of the long noncoding RNA-LET by histone deacetylase 3 contributes to hypoxia-mediated metastasis. Mol Cell.

[39] Liu YW, Sun M, Xia R, Zhang EB, Liu XH, Zhang ZH, Xu TP, De W, Liu BR, Wang ZX (2015). LincHOTAIR epigenetically silences miR34a by binding to PRC2 to promote the epithelial-to-mesenchymal transition in human gastric cancer. Cell Death Dis.

[40] Tani H, Torimura M, Akimitsu N (2013). The RNA degradation pathway regulates the function of GAS5 a non-coding RNA in mammalian cells. PLoS One.

[41] Kino T, Hurt DE, Ichijo T, Nader N, Chrousos GP (2010). Noncoding RNA gas5 is a growth arrest- and starvation-associated repressor of the glucocorticoid receptor. Sci Signal.

[42] Ma MZ, Chu BF, Zhang Y, Weng MZ, Qin YY, Gong W, Quan ZW (2015). Long non-coding RNA CCAT1 promotes gallbladder cancer development via negative modulation of miRNA-218-5p. Cell Death Dis.

[43] Cai M, Wang Z, Zhang J, Zhou H, Jin L, Bai R, Weng Y (2015). Adam17, a Target of Mir-326, Promotes Emt-Induced Cells Invasion in Lung Adenocarcinoma. Cell Physiol Biochem.

[44] Sun C, Huang C, Li S, Yang C, Xi Y, Wang L, Zhang F, Fu Y, Li D (2016). Hsa-miR-326 targets CCND1 and inhibits non-small cell lung cancer development. Oncotarget.

[45] Wang R, Chen X, Xu T, Xia R, Han L, Chen W, De W, Shu Y (2016). MiR-326 regulates cell proliferation and migration in lung cancer by targeting phox2a and is regulated by HOTAIR. Am J Cancer Res.

[46] Li J, Li S, Chen Z, Wang J, Chen Y, Xu Z, Jin M, Yu W (2016). miR-326 reverses chemoresistance in human lung adenocarcinoma cells by targeting specificity protein 1. Tumour Biol.

[47] Liang Z, Wu H, Xia J, Li Y, Zhang Y, Huang K, Wagar N, Yoon Y, Cho HT, Scala S, Shim H (2010). Involvement of miR-326 in chemotherapy resistance of breast cancer through modulating expression of multidrug resistance-associated protein 1. Biochem Pharmacol.

[48] Su BB, Zhou SW, Gan CB, Zhang XN (2016). MiR-330-5p regulates tyrosinase and PDIA3 expression and suppresses cell proliferation and invasion in cutaneous malignant melanoma. J Surg Res.

[49] Li Y, Zhu X, Xu W, Wang D, Yan J (2013). miR-330 regulates the proliferation of colorectal cancer cells by targeting Cdc42. Biochem Biophys Res Commun.

[50] Qiu S, Lin S, Hu D, Feng Y, Tan Y, Peng Y (2013). Interactions of miR-323/miR-326/miR-329 and miR-130a/miR-155/miR-210 as prognostic indicators for clinical outcome of glioblastoma patients. J Transl Med.

[51] Yao Y, Xue Y, Ma J, Shang C, Wang P, Liu L, Liu W, Li Z, Qu S, Li Z, Liu Y (2014). MiR-330-mediated regulation of SH3GL2 expression enhances malignant behaviors of glioblastoma stem cells by activating ERK and PI3K/AKT signaling pathways. PLoS One.

[52] Kim BK, Yoo HI, Choi K, Yoon SK (2015). miR-330-5p inhibits proliferation and migration of keratinocytes by targeting Pdia3 expression. FEBS J.

[53] Li J, Li S, Chen Z, Wang J, Chen Y, Xu Z, Jin M, Yu W (2016). miR-326 reverses chemoresistance in human lung adenocarcinoma cells by targeting specificity protein 1. Tumour Biol.

[54] Wang R, Chen X, Xu T, Xia R, Han L, Chen W, De W, Shu Y (2016). MiR-326 regulates cell proliferation and migration in lung cancer by targeting phox2a and is regulated by HOTAIR. Am J Cancer Res.

[55] Chen J, Bai M, Ning C, Xie B, Zhang J, Liao H, Xiong J, Tao X, Yan D, Xi X, Chen X, Yu Y, Bast RC (2016). Gankyrin facilitates follicle-stimulating hormone-driven ovarian cancer cell proliferation through the PI3K/AKT/HIF-1α/cyclin D1 pathway. Oncogene.

[56] Fusté NP, Castelblanco E, Felip I, Santacana M, Fernández-Hernández R, Gatius S, Pedraza N, Pallarés J, Cemeli T, Valls J, Tarres M, Ferrezuelo F, Dolcet X (2016). Characterization of cytoplasmic cyclin D1 as a marker of invasiveness in cancer. Oncotarget.

[57] Lee Y, Ko D, Min HJ, Kim SB, Ahn HM, Lee Y, Kim S (2016). TMPRSS4 induces invasion and proliferation of prostate cancer cells through induction of Slug and cyclin D1. Oncotarget.

[58] Marampon F, Gravina G, Ju X, Vetuschi A, Sferra R, Casimiro M, Pompili S, Festuccia C, Colapietro A, Gaudio E, Di Cesare E, Tombolini V, Pestell RG (2016). Cyclin D1 silencing suppresses tumorigenicity, impairs DNA double strand break repair and thus radiosensitizes androgen-independent prostate cancer cells to DNA damage. Oncotarget.

[59] Yang P, Chen W, Li X, Eilers G, He Q, Liu L, Wu Y, Wu Y, Yu W, Fletcher JA, Ou WB (2016). Downregulation of cyclin D1 sensitizes cancer cells to MDM2 antagonist Nutlin-3. Oncotarget.

[60] Kennedy AL, Vallurupalli M, Chen L, Crompton B, Cowley G, Vazquez F, Weir BA, Tsherniak A, Parasuraman S, Kim S, Alexe G, Stegmaier K (2015). Functional, chemical genomic, and super-enhancer screening identify sensitivity to cyclin D1/CDK4 pathway inhibition in Ewing sarcoma. Oncotarget.

[61] Casimiro MC, Di Sante G, Crosariol M, Loro E, Dampier W, Ertel A, Yu Z, Saria EA, Papanikolaou A, Li Z, Wang C, Addya S, Lisanti MP (2015). Kinase-independent role of cyclin D1 in chromosomal instability and mammary tumorigenesis. Oncotarget.

[62] Hwang SJ, Lee HW, Kim HR, Song HJ, Lee DH, Lee H, Shin CH, Joung JG, Kim DH, Joo KM, Kim HH (2015). Overexpression of microRNA-95-3p suppresses brain metastasis of lung adenocarcinoma through downregulation of cyclin D1. Oncotarget.

