# Supplementary material for: Long non-coding RNA EWSAT1 promotes human nasopharyngeal carcinoma cell growth in vitro by targeting miR-326/-330-5p
Source: Aging (Albany NY). 2016 Nov 3;8(11):2948–59. doi: 10.18632/aging.101103 (PMC5182074; doi:10.18632/aging.101103)
Supplement: Supplementary file 1 [file aging-08-2948-s001.pdf]

SUPPLEMENTARY MATERIAL

Table S1. Predicted results using miRDB (target score≥50)

Target Detail	Target Rank	Target Score	miRNA Name	Gene Symbol
	1	92	hsa-miR-4296	submission
	2	87	hsa-miR-4291	submission
	3	81	hsa-miR-3168	submission
	4	79	hsa-miR-5004-5p	submission
	5	78	hsa-miR-1224-5p	submission
	6	76	hsa-miR-4322	submission
	7	76	hsa-miR-4265	submission
	8	76	hsa-miR-3713	submission
	9	75	hsa-miR-4516	submission
	10	71	hsa-miR-497-3p	submission
	11	69	hsa-miR-27a-5p	submission
	12	69	hsa-miR-3192-5p	submission
	13	69	hsa-miR-6825-5p	submission
	14	66	hsa-miR-8065	submission
	15	65	hsa-miR-4793-5p	submission
	16	64	hsa-miR-6875-5p	submission
	17	64	hsa-miR-3126-5p	submission
	18	64	hsa-miR-4534	submission
	19	62	hsa-miR-4459	submission
	20	61	hsa-miR-4316	submission
	21	60	hsa-miR-922	submission
	22	60	hsa-miR-6891-5p	submission
	23	58	hsa-miR-330-5p	submission
	24	58	hsa-miR-326	submission
	25	57	hsa-miR-4426	submission
	26	56	hsa-miR-5195-3p	submission
	27	55	hsa-miR-4293	submission
	28	55	hsa-miR-6868-5p	submission
	29	55	hsa-miR-1296-3p	submission
	30	54	hsa-miR-3187-3p	submission
	31	54	hsa-miR-4779	submission
	32	53	hsa-miR-581	submission
	33	53	hsa-miR-4685-3p	submission
	34	53	hsa-miR-4287	submission
	35	52	hsa-miR-3120-3p	submission
	36	51	hsa-miR-6765-3p	submission
	37	50	hsa-miR-4802-5p	submission
	38	50	hsa-miR-4483	submission

Please browse the Full Text version of this manuscript to see **Table S2. Predicted results using PITA (target score≤-20).**
