# Supplementary material for: Long non-coding RNA EWSAT1 promotes human nasopharyngeal carcinoma cell growth in vitro by targeting miR-326/-330-5p
Source: Aging (Albany NY). 2016 Nov 3;8(11):2948–59. doi: 10.18632/aging.101103 (PMC5182074; doi:10.18632/aging.101103)
Supplement: Supplementary file 2 [file aging-08-2948-s002.pdf]

Predicted results using PITA (target score≤-20)

Gene	microRNA	Sites	Score
Seq1	hsa-miR-671-5p	4	-31
Seq1	hsa-miR-608	14	-30.31
Seq1	hsa-miR-330-5p	6	-30.3
Seq1	hsa-miR-661	8	-27.41
Seq1	hsa-miR-324-5p	4	-27.2
Seq1	hsa-miR-326	6	-26.8
Seq1	hsa-miR-296-3p	11	-26.63
Seq1	hsa-miR-1182	12	-26.45
Seq1	hsa-miR-502-3p	8	-24.8
Seq1	hsa-miR-657	5	-24.62
Seq1	hsa-miR-939	7	-24.62
Seq1	hsa-miR-1254	4	-24.6
Seq1	hsa-miR-638	3	-24.1
Seq1	hsa-miR-1237	8	-23.61
Seq1	hsa-miR-367	2	-23.3
Seq1	hsa-miR-612	5	-23.2
Seq1	hsa-miR-1226	4	-22.7
Seq1	hsa-miR-1268	7	-22.66
Seq1	hsa-miR-760	11	-22.64
Seq1	hsa-miR-766	7	-22.23
Seq1	hsa-miR-373	9	-21.99
Seq1	hsa-miR-520e	9	-21.8
Seq1	hsa-miR-198	8	-21.6
Seq1	hsa-miR-323-5p	5	-21.5
Seq1	hsa-miR-188-5p	5	-21.47
Seq1	hsa-miR-423-5p	9	-21.45
Seq1	hsa-miR-422a	4	-21.33
Seq1	hsa-miR-1231	13	-21.22
Seq1	hsa-miR-26a	4	-21.2
Seq1	hsa-miR-139-5p	5	-21.15
Seq1	hsa-miR-342-5p	10	-21.1
Seq1	hsa-miR-601	6	-20.92
Seq1	hsa-miR-1234	5	-20.9
Seq1	hsa-miR-675	9	-20.84
Seq1	hsa-miR-302c	9	-20.81
Seq1	hsa-miR-361-3p	5	-20.8
Seq1	hsa-miR-582-5p	6	-20.8
Seq1	hsa-miR-510	3	-20.7
Seq1	hsa-miR-520f	10	-20.64
Seq1	hsa-miR-520d-3p	9	-20.56
Seq1	hsa-miR-127-5p	3	-20.5
Seq1	hsa-miR-1266	13	-20.45
Seq1	hsa-miR-544	10	-20.2
Seq1	hsa-miR-1294	11	-20.17
Seq1	hsa-miR-658	13	-20.17

Seq1	hsa-miR-135b	7	-20.14
Seq1	hsa-miR-145	13	-20.11
Seq1	hsa-miR-1260	4	-20.05
Seq1	hsa-miR-663	5	-20.05
Seq1	hsa-miR-765	15	-20.03
Seq1	hsa-miR-1308	10	-20
Seq1	hsa-miR-302e	9	-19.9
Seq1	hsa-miR-92a	2	-19.9
Seq1	hsa-miR-1293	9	-19.78
Seq1	hsa-miR-942	9	-19.74
Seq1	hsa-miR-768-5p	12	-19.64
Seq1	hsa-miR-223	7	-19.59
Seq1	hsa-miR-1274b	6	-19.55
Seq1	hsa-miR-34a	7	-19.41
Seq1	hsa-miR-1274a	5	-19.38
Seq1	hsa-miR-933	3	-19.32
Seq1	hsa-miR-507	5	-19.3
Seq1	hsa-miR-770-5p	3	-19.3
Seq1	hsa-miR-92b	2	-19.3
Seq1	hsa-miR-525-5p	8	-19.22
Seq1	hsa-let-7e	8	-19.05
Seq1	hsa-miR-1321	12	-19.01
Seq1	hsa-miR-609	9	-19
Seq1	hsa-miR-302a	9	-18.93
Seq1	hsa-miR-557	5	-18.92
Seq1	hsa-miR-1265	7	-18.87
Seq1	hsa-miR-654-5p	3	-18.87
Seq1	hsa-miR-1207-5p	8	-18.85
Seq1	hsa-miR-152	4	-18.81
Seq1	hsa-miR-502-5p	11	-18.81
Seq1	hsa-miR-1208	2	-18.8
Seq1	hsa-miR-520d-5p	7	-18.76
Seq1	hsa-miR-518a-5p	12	-18.73
Seq1	hsa-miR-527	12	-18.73
Seq1	hsa-miR-378	4	-18.72
Seq1	hsa-miR-520c-3p	9	-18.71
Seq1	hsa-miR-623	6	-18.71
Seq1	hsa-miR-302b	9	-18.7
Seq1	hsa-miR-520b	9	-18.7
Seq1	hsa-miR-1280	3	-18.68
Seq1	hsa-miR-937	4	-18.67
Seq1	hsa-miR-105	4	-18.6
Seq1	hsa-miR-26b	4	-18.6
Seq1	hsa-miR-24	7	-18.51
Seq1	hsa-miR-302d	9	-18.45
Seq1	hsa-miR-516a-3p	8	-18.4
Seq1	hsa-miR-520a-5p	8	-18.4

Seq1	hsa-miR-1236	10	-18.37
Seq1	hsa-miR-135a	7	-18.34
Seq1	hsa-miR-449b	7	-18.33
Seq1	hsa-miR-339-5p	2	-18.32
Seq1	hsa-miR-449a	7	-18.22
Seq1	hsa-miR-1178	4	-18.21
Seq1	hsa-miR-1258	4	-18.17
Seq1	hsa-miR-1249	5	-18.11
Seq1	hsa-miR-1233	8	-18.09
Seq1	hsa-miR-659	3	-18
Seq1	hsa-miR-1183	1	-17.94
Seq1	hsa-miR-202	3	-17.94
Seq1	hsa-miR-671-3p	6	-17.89
Seq1	hsa-miR-193a-5p	16	-17.85
Seq1	hsa-miR-216b	5	-17.8
Seq1	hsa-miR-566	3	-17.7
Seq1	hsa-miR-936	8	-17.7
Seq1	hsa-miR-520g	2	-17.65
Seq1	hsa-miR-491-5p	7	-17.54
Seq1	hsa-miR-520a-3p	9	-17.53
Seq1	hsa-miR-204	8	-17.46
Seq1	hsa-miR-532-5p	4	-17.46
Seq1	hsa-miR-211	8	-17.4
Seq1	hsa-miR-518e	6	-17.4
Seq1	hsa-miR-1225-3p	5	-17.37
Seq1	hsa-miR-491-3p	5	-17.23
Seq1	hsa-miR-541	8	-17.13
Seq1	hsa-miR-25	2	-17.1
Seq1	hsa-miR-1248	11	-17.05
Seq1	hsa-miR-217	2	-17
Seq1	hsa-miR-501-3p	5	-17
Seq1	hsa-miR-614	2	-16.99
Seq1	hsa-miR-299-3p	9	-16.91
Seq1	hsa-miR-331-3p	3	-16.9
Seq1	hsa-miR-644	8	-16.9
Seq1	hsa-miR-885-3p	3	-16.87
Seq1	hsa-miR-125a-3p	7	-16.7
Seq1	hsa-miR-1322	4	-16.7
Seq1	hsa-miR-1323	2	-16.7
Seq1	hsa-miR-1275	13	-16.68
Seq1	hsa-miR-1229	3	-16.62
Seq1	hsa-miR-93	4	-16.6
Seq1	hsa-miR-485-5p	8	-16.4
Seq1	hsa-miR-654-3p	3	-16.39
Seq1	hsa-miR-412	5	-16.38
Seq1	hsa-let-7b	6	-16.36
Seq1	hsa-miR-140-5p	8	-16.33

Seq1	hsa-miR-503	4	-16.31
Seq1	hsa-miR-634	4	-16.22
Seq1	hsa-miR-431	9	-16.12
Seq1	hsa-miR-877	11	-16.09
Seq1	hsa-miR-500	6	-16.06
Seq1	hsa-miR-1224-5p	6	-16.05
Seq1	hsa-miR-197	4	-16.04
Seq1	hsa-miR-519b-3p	5	-15.98
Seq1	hsa-miR-1205	8	-15.94
Seq1	hsa-miR-34c-5p	7	-15.91
Seq1	hsa-miR-220b	3	-15.83
Seq1	hsa-miR-602	3	-15.67
Seq1	hsa-miR-1292	2	-15.62
Seq1	hsa-miR-148b	4	-15.62
Seq1	hsa-miR-513a-5p	8	-15.61
Seq1	hsa-miR-876-3p	10	-15.61
Seq1	hsa-miR-1306	4	-15.55
Seq1	hsa-miR-148a	5	-15.52
Seq1	hsa-miR-1224-3p	3	-15.47
Seq1	hsa-miR-199a-5p	6	-15.4
Seq1	hsa-miR-629	4	-15.35
Seq1	hsa-miR-596	2	-15.34
Seq1	hsa-miR-138	3	-15.3
Seq1	hsa-miR-513c	6	-15.22
Seq1	hsa-miR-106b	4	-15.2
Seq1	hsa-miR-501-5p	7	-15.2
Seq1	hsa-miR-940	12	-15.17
Seq1	hsa-miR-1246	11	-15.14
Seq1	hsa-miR-106a	4	-15.1
Seq1	hsa-miR-17	4	-15.1
Seq1	hsa-miR-20a	4	-15.1
Seq1	hsa-miR-452	5	-15.1
Seq1	hsa-miR-520h	2	-15.1
Seq1	hsa-miR-615-5p	2	-15.08
Seq1	hsa-miR-372	3	-15.06
Seq1	hsa-miR-27b	4	-14.92
Seq1	hsa-miR-205	7	-14.91
Seq1	hsa-miR-515-3p	4	-14.85
Seq1	hsa-miR-453	4	-14.84
Seq1	hsa-miR-362-5p	4	-14.82
Seq1	hsa-miR-1290	7	-14.81
Seq1	hsa-miR-1184	8	-14.8
Seq1	hsa-miR-576-5p	8	-14.75
Seq1	hsa-miR-409-5p	2	-14.7
Seq1	hsa-miR-1303	5	-14.67
Seq1	hsa-miR-648	5	-14.67
Seq1	hsa-miR-33b	4	-14.65

Seq1	hsa-miR-432	5	-14.62
Seq1	hsa-miR-346	1	-14.5
Seq1	hsa-miR-448	10	-14.49
Seq1	hsa-miR-619	4	-14.45
Seq1	hsa-miR-886-3p	7	-14.44
Seq1	hsa-miR-375	5	-14.41
Seq1	hsa-miR-631	1	-14.4
Seq1	hsa-miR-298	10	-14.38
Seq1	hsa-miR-516b	10	-14.36
Seq1	hsa-miR-1270	8	-14.35
Seq1	hsa-miR-184	9	-14.3
Seq1	hsa-miR-382	4	-14.3
Seq1	hsa-miR-1255b	2	-14.29
Seq1	hsa-miR-744	5	-14.29
Seq1	hsa-miR-576-3p	10	-14.24
Seq1	hsa-miR-571	7	-14.22
Seq1	hsa-miR-20b	4	-14.2
Seq1	hsa-miR-921	6	-14.15
Seq1	hsa-miR-941	2	-14.14
Seq1	hsa-miR-27a	4	-14.1
Seq1	hsa-miR-1301	7	-14.06
Seq1	hsa-miR-524-5p	7	-14.06
Seq1	hsa-miR-511	11	-14.02
Seq1	hsa-miR-664	9	-13.9
Seq1	hsa-miR-519a	5	-13.89
Seq1	hsa-miR-635	8	-13.87
Seq1	hsa-miR-1299	6	-13.84
Seq1	hsa-miR-210	8	-13.84
Seq1	hsa-miR-190b	8	-13.83
Seq1	hsa-miR-187	5	-13.8
Seq1	hsa-miR-421	2	-13.75
Seq1	hsa-miR-450a	5	-13.75
Seq1	hsa-miR-1304	13	-13.72
Seq1	hsa-let-7c	6	-13.7
Seq1	hsa-miR-151-3p	4	-13.7
Seq1	hsa-miR-96	1	-13.7
Seq1	hsa-miR-122	6	-13.68
Seq1	hsa-miR-455-3p	6	-13.68
Seq1	hsa-miR-662	1	-13.62
Seq1	hsa-let-7d	6	-13.59
Seq1	hsa-miR-28-5p	6	-13.57
Seq1	hsa-miR-134	5	-13.5
Seq1	hsa-miR-149	5	-13.48
Seq1	hsa-miR-450b-5p	3	-13.4
Seq1	hsa-miR-550	5	-13.36
Seq1	hsa-miR-147b	3	-13.32
Seq1	hsa-miR-665	7	-13.31

Seq1	hsa-miR-19b	5	-13.27
Seq1	hsa-miR-497	6	-13.23
Seq1	hsa-miR-607	4	-13.16
Seq1	hsa-miR-450b-3p	5	-13.13
Seq1	hsa-miR-637	7	-13.13
Seq1	hsa-miR-943	5	-13.13
Seq1	hsa-miR-34c-3p	6	-13.12
Seq1	hsa-miR-567	5	-13.01
Seq1	hsa-miR-1297	4	-13
Seq1	hsa-miR-493	7	-12.9
Seq1	hsa-miR-617	2	-12.9
Seq1	hsa-miR-519c-3p	5	-12.85
Seq1	hsa-miR-616	7	-12.84
Seq1	hsa-miR-181a	5	-12.83
Seq1	hsa-miR-370	3	-12.82
Seq1	hsa-miR-554	1	-12.82
Seq1	hsa-miR-196a	4	-12.75
Seq1	hsa-miR-876-5p	6	-12.74
Seq1	hsa-miR-578	7	-12.72
Seq1	hsa-miR-1255a	2	-12.69
Seq1	hsa-miR-483-5p	4	-12.69
Seq1	hsa-miR-647	4	-12.65
Seq1	hsa-miR-181c	5	-12.64
Seq1	hsa-miR-1302	5	-12.6
Seq1	hsa-miR-486-3p	6	-12.51
Seq1	hsa-miR-1279	2	-12.5
Seq1	hsa-miR-489	3	-12.5
Seq1	hsa-miR-33a	7	-12.49
Seq1	hsa-miR-708	7	-12.49
Seq1	hsa-miR-517a	5	-12.45
Seq1	hsa-miR-182	4	-12.4
Seq1	hsa-miR-938	2	-12.4
Seq1	hsa-miR-572	2	-12.39
Seq1	hsa-miR-922	8	-12.33
Seq1	hsa-miR-331-5p	3	-12.31
Seq1	hsa-miR-517c	5	-12.3
Seq1	hsa-miR-196b	4	-12.27
Seq1	hsa-miR-490-3p	4	-12.26
Seq1	hsa-miR-522	7	-12.16
Seq1	hsa-miR-302f	6	-12.1
Seq1	hsa-miR-129-5p	5	-12.01
Seq1	hsa-miR-129-3p	4	-12
Seq1	hsa-miR-512-3p	7	-11.96
Seq1	hsa-miR-1203	5	-11.95
Seq1	hsa-miR-526b	8	-11.93
Seq1	hsa-miR-9	6	-11.93
Seq1	hsa-miR-206	3	-11.82

Seq1	hsa-miR-34b	4	-11.82
Seq1	hsa-miR-1286	3	-11.74
Seq1	hsa-let-7a	6	-11.71
Seq1	hsa-miR-423-3p	1	-11.7
Seq1	hsa-miR-153	5	-11.65
Seq1	hsa-miR-30b	5	-11.65
Seq1	hsa-miR-455-5p	8	-11.64
Seq1	hsa-miR-219-1-3p	5	-11.63
Seq1	hsa-miR-136	12	-11.56
Seq1	hsa-miR-219-2-3p	4	-11.56
Seq1	hsa-miR-630	8	-11.56
Seq1	hsa-miR-30e	5	-11.53
Seq1	hsa-miR-584	4	-11.5
Seq1	hsa-miR-920	5	-11.48
Seq1	hsa-miR-513a-3p	5	-11.4
Seq1	hsa-miR-345	4	-11.38
Seq1	hsa-miR-409-3p	3	-11.34
Seq1	hsa-miR-186	4	-11.31
Seq1	hsa-miR-199b-5p	6	-11.3
Seq1	hsa-miR-190	8	-11.26
Seq1	hsa-miR-650	10	-11.25
Seq1	hsa-miR-1298	4	-11.2
Seq1	hsa-let-7f	6	-11.17
Seq1	hsa-miR-297	17	-11.17
Seq1	hsa-miR-587	7	-11.15
Seq1	hsa-miR-1271	3	-11.1
Seq1	hsa-miR-551a	1	-11.1
Seq1	hsa-miR-30a	5	-11.09
Seq1	hsa-miR-1180	10	-11
Seq1	hsa-miR-133a	3	-11
Seq1	hsa-miR-19a	5	-10.97
Seq1	hsa-miR-548g	3	-10.97
Seq1	hsa-miR-593	9	-10.89
Seq1	hsa-miR-1202	5	-10.84
Seq1	hsa-miR-484	5	-10.83
Seq1	hsa-miR-552	5	-10.69
Seq1	hsa-miR-133b	3	-10.67
Seq1	hsa-miR-579	7	-10.65
Seq1	hsa-miR-30c	5	-10.63
Seq1	hsa-miR-642	6	-10.6
Seq1	hsa-miR-516a-5p	5	-10.56
Seq1	hsa-miR-1256	7	-10.5
Seq1	hsa-miR-103	4	-10.47
Seq1	hsa-miR-218	14	-10.45
Seq1	hsa-miR-194	3	-10.44
Seq1	hsa-miR-886-5p	3	-10.42
Seq1	hsa-miR-655	3	-10.41

Seq1	hsa-miR-1305	6	-10.4
Seq1	hsa-miR-139-3p	2	-10.4
Seq1	hsa-miR-515-5p	4	-10.36
Seq1	hsa-miR-555	1	-10.3
Seq1	hsa-miR-599	6	-10.3
Seq1	hsa-miR-621	1	-10.3
Seq1	hsa-miR-220c	4	-10.23
Seq1	hsa-miR-628-3p	3	-10.21
Seq1	hsa-miR-632	2	-10.11
Seq1	hsa-miR-486-5p	3	-10.1
Seq1	hsa-let-7i	6	-10.09
Seq1	hsa-miR-574-5p	3	-10.01
Seq1	hsa-miR-1244	5	-9.98
Seq1	hsa-miR-214	3	-9.98
Seq1	hsa-miR-645	4	-9.96
Seq1	hsa-miR-185	6	-9.95
Seq1	hsa-miR-923	7	-9.92
Seq1	hsa-miR-1	3	-9.9
Seq1	hsa-miR-509-3p	4	-9.9
Seq1	hsa-miR-873	9	-9.87
Seq1	hsa-miR-646	2	-9.77
Seq1	hsa-miR-651	3	-9.77
Seq1	hsa-miR-1273	4	-9.71
Seq1	hsa-miR-1200	6	-9.68
Seq1	hsa-miR-363	6	-9.68
Seq1	hsa-miR-924	5	-9.68
Seq1	hsa-miR-490-5p	3	-9.63
Seq1	hsa-miR-1207-3p	4	-9.62
Seq1	hsa-miR-95	2	-9.62
Seq1	hsa-miR-574-3p	3	-9.61
Seq1	hsa-miR-643	11	-9.61
Seq1	hsa-miR-521	2	-9.6
Seq1	hsa-miR-1289	7	-9.55
Seq1	hsa-miR-18a	3	-9.54
Seq1	hsa-miR-101	2	-9.5
Seq1	hsa-miR-625	7	-9.5
Seq1	hsa-miR-495	2	-9.41
Seq1	hsa-miR-548l	8	-9.4
Seq1	hsa-miR-626	4	-9.39
Seq1	hsa-miR-506	2	-9.38
Seq1	hsa-miR-487a	3	-9.36
Seq1	hsa-miR-16	6	-9.35
Seq1	hsa-miR-548c-3p	2	-9.35
Seq1	hsa-miR-1281	3	-9.31
Seq1	hsa-miR-558	4	-9.31
Seq1	hsa-miR-887	2	-9.28
Seq1	hsa-miR-107	4	-9.24

Seq1	hsa-miR-588	2	-9.23
Seq1	hsa-miR-604	7	-9.23
Seq1	hsa-miR-362-3p	4	-9.22
Seq1	hsa-miR-125b	3	-9.19
Seq1	hsa-miR-1282	2	-9.17
Seq1	hsa-miR-636	6	-9.17
Seq1	hsa-miR-18b	3	-9.16
Seq1	hsa-miR-627	5	-9.14
Seq1	hsa-miR-569	6	-9.13
Seq1	hsa-miR-545	4	-9.1
Seq1	hsa-miR-23b	5	-9.09
Seq1	hsa-miR-622	4	-9.09
Seq1	hsa-miR-1300	1	-9.03
Seq1	hsa-miR-518d-5p	4	-9.03
Seq1	hsa-miR-520c-5p	4	-9.03
Seq1	hsa-miR-526a	4	-9.03
Seq1	hsa-miR-30d	5	-9
Seq1	hsa-miR-874	4	-8.93
Seq1	hsa-miR-769-3p	5	-8.89
Seq1	hsa-miR-1259	8	-8.85
Seq1	hsa-miR-7	4	-8.85
Seq1	hsa-miR-1825	3	-8.8
Seq1	hsa-miR-128	4	-8.75
Seq1	hsa-miR-595	3	-8.75
Seq1	hsa-miR-610	4	-8.73
Seq1	hsa-miR-499-3p	3	-8.7
Seq1	hsa-miR-767-3p	6	-8.69
Seq1	hsa-miR-508-5p	3	-8.67
Seq1	hsa-miR-519b-5p	4	-8.63
Seq1	hsa-miR-519c-5p	4	-8.63
Seq1	hsa-miR-580	5	-8.63
Seq1	hsa-miR-575	6	-8.61
Seq1	hsa-miR-483-3p	3	-8.53
Seq1	hsa-miR-556-3p	3	-8.53
Seq1	hsa-miR-220a	3	-8.39
Seq1	hsa-miR-338-3p	3	-8.39
Seq1	hsa-miR-142-3p	3	-8.36
Seq1	hsa-miR-1247	2	-8.35
Seq1	hsa-miR-1295	2	-8.32
Seq1	hsa-miR-1264	2	-8.26
Seq1	hsa-miR-15a	6	-8.26
Seq1	hsa-miR-720	6	-8.23
Seq1	hsa-miR-203	3	-8.21
Seq1	hsa-miR-548o	2	-8.21
Seq1	hsa-miR-1827	9	-8.16
Seq1	hsa-miR-1238	3	-8.1
Seq1	hsa-miR-1267	1	-8.1

Seq1	hsa-miR-144	4	-8.09
Seq1	hsa-miR-892a	5	-8.09
Seq1	hsa-miR-1253	5	-8.07
Seq1	hsa-miR-1251	7	-8.02
Seq1	hsa-miR-137	4	-7.98
Seq1	hsa-miR-32	6	-7.94
Seq1	hsa-miR-573	3	-7.84
Seq1	hsa-miR-195	6	-7.8
Seq1	hsa-miR-556-5p	4	-7.76
Seq1	hsa-let-7g	6	-7.72
Seq1	hsa-miR-365	2	-7.7
Seq1	hsa-miR-539	4	-7.69
Seq1	hsa-miR-98	6	-7.68
Seq1	hsa-miR-125a-5p	3	-7.65
Seq1	hsa-miR-23a	5	-7.6
Seq1	hsa-miR-592	5	-7.6
Seq1	hsa-miR-663b	1	-7.52
Seq1	hsa-miR-499-5p	2	-7.5
Seq1	hsa-miR-583	3	-7.35
Seq1	hsa-miR-1225-5p	1	-7.34
Seq1	hsa-miR-1826	3	-7.31
Seq1	hsa-miR-548f	2	-7.3
Seq1	hsa-miR-581	7	-7.3
Seq1	hsa-miR-1257	2	-7.2
Seq1	hsa-miR-15b	6	-7.14
Seq1	hsa-miR-181b	4	-7.06
Seq1	hsa-miR-548k	6	-7.02
Seq1	hsa-miR-1204	3	-6.99
Seq1	hsa-miR-329	4	-6.94
Seq1	hsa-miR-1206	9	-6.93
Seq1	hsa-miR-150	2	-6.93
Seq1	hsa-miR-488	1	-6.92
Seq1	hsa-miR-1276	2	-6.89
Seq1	hsa-miR-1287	1	-6.88
Seq1	hsa-miR-1245	6	-6.76
Seq1	hsa-miR-543	1	-6.72
Seq1	hsa-miR-224	2	-6.64
Seq1	hsa-miR-1228	3	-6.48
Seq1	hsa-miR-31	3	-6.48
Seq1	hsa-miR-1250	4	-6.45
Seq1	hsa-miR-585	3	-6.4
Seq1	hsa-miR-193a-3p	1	-6.39
Seq1	hsa-miR-523	1	-6.31
Seq1	hsa-miR-769-5p	4	-6.3
Seq1	hsa-miR-605	3	-6.24
Seq1	hsa-miR-208a	3	-6.14
Seq1	hsa-miR-582-3p	4	-6.12

Seq1	hsa-miR-146b-3p	2	-6.1
Seq1	hsa-miR-1288	2	-6.08
Seq1	hsa-miR-132	4	-6.04
Seq1	hsa-miR-590-3p	3	-6
Seq1	hsa-miR-568	3	-5.96
Seq1	hsa-miR-618	3	-5.96
Seq1	hsa-miR-424	10	-5.94
Seq1	hsa-miR-496	2	-5.92
Seq1	hsa-miR-425	1	-5.86
Seq1	hsa-miR-1262	3	-5.83
Seq1	hsa-miR-146a	4	-5.83
Seq1	hsa-miR-641	2	-5.82
Seq1	hsa-miR-140-3p	2	-5.79
Seq1	hsa-miR-606	2	-5.7
Seq1	hsa-miR-512-5p	4	-5.68
Seq1	hsa-miR-802	2	-5.65
Seq1	hsa-miR-376a	6	-5.63
Seq1	hsa-miR-376b	6	-5.63
Seq1	hsa-miR-199a-3p	3	-5.59
Seq1	hsa-miR-199b-3p	3	-5.59
Seq1	hsa-miR-600	6	-5.59
Seq1	hsa-miR-28-3p	5	-5.56
Seq1	hsa-miR-222	1	-5.54
Seq1	hsa-miR-549	2	-5.52
Seq1	hsa-miR-221	1	-5.49
Seq1	hsa-miR-296-5p	3	-5.47
Seq1	hsa-miR-590-5p	3	-5.47
Seq1	hsa-miR-1201	4	-5.41
Seq1	hsa-miR-147	5	-5.4
Seq1	hsa-miR-181d	4	-5.4
Seq1	hsa-miR-146b-5p	4	-5.39
Seq1	hsa-miR-620	8	-5.34
Seq1	hsa-miR-518c	5	-5.33
Seq1	hsa-miR-1291	3	-5.21
Seq1	hsa-miR-548i	6	-5.2
Seq1	hsa-miR-649	2	-5.17
Seq1	hsa-miR-514	2	-5.11
Seq1	hsa-miR-1261	2	-5.07
Seq1	hsa-miR-505	1	-5.05
Seq1	hsa-miR-548b-5p	6	-5.05
Seq1	hsa-miR-1197	4	-5.03
Seq1	hsa-miR-342-3p	2	-4.97
Seq1	hsa-miR-154	3	-4.94
Seq1	hsa-miR-518d-3p	5	-4.88
Seq1	hsa-miR-1269	2	-4.83
Seq1	hsa-miR-892b	3	-4.83
Seq1	hsa-miR-524-3p	2	-4.8

Seq1	hsa-miR-944	1	-4.65
Seq1	hsa-miR-337-5p	4	-4.62
Seq1	hsa-miR-542-3p	5	-4.57
Seq1	hsa-miR-299-5p	2	-4.56
Seq1	hsa-miR-591	4	-4.49
Seq1	hsa-miR-548n	6	-4.48
Seq1	hsa-miR-377	3	-4.45
Seq1	hsa-miR-379	3	-4.45
Seq1	hsa-miR-381	1	-4.44
Seq1	hsa-miR-548j	6	-4.43
Seq1	hsa-miR-561	3	-4.35
Seq1	hsa-miR-624	4	-4.35
Seq1	hsa-miR-1296	2	-4.3
Seq1	hsa-miR-1263	4	-4.29
Seq1	hsa-miR-212	4	-4.29
Seq1	hsa-miR-361-5p	2	-4.26
Seq1	hsa-miR-328	1	-4.21
Seq1	hsa-miR-143	4	-4.17
Seq1	hsa-miR-1285	1	-4.12
Seq1	hsa-miR-525-3p	2	-4.04
Seq1	hsa-miR-1179	5	-4.01
Seq1	hsa-miR-29b	2	-4.01
Seq1	hsa-miR-548e	1	-4
Seq1	hsa-miR-934	4	-4
Seq1	hsa-miR-551b	2	-3.95
Seq1	hsa-miR-219-5p	3	-3.91
Seq1	hsa-miR-589	1	-3.9
Seq1	hsa-miR-339-3p	2	-3.89
Seq1	hsa-miR-660	1	-3.86
Seq1	hsa-miR-548p	4	-3.84
Seq1	hsa-miR-518a-3p	5	-3.83
Seq1	hsa-miR-563	3	-3.83
Seq1	hsa-miR-1252	7	-3.71
Seq1	hsa-miR-875-3p	2	-3.7
Seq1	hsa-miR-191	1	-3.52
Seq1	hsa-miR-509-5p	2	-3.51
Seq1	hsa-miR-1243	2	-3.49
Seq1	hsa-miR-323-3p	3	-3.42
Seq1	hsa-miR-335	8	-3.42
Seq1	hsa-miR-330-3p	2	-3.41
Seq1	hsa-miR-10a	3	-3.35
Seq1	hsa-miR-517b	3	-3.34
Seq1	hsa-miR-509-3-5p	2	-3.29
Seq1	hsa-miR-548b-3p	2	-3.28
Seq1	hsa-miR-183	2	-3.23
Seq1	hsa-miR-518f	5	-3.22
Seq1	hsa-miR-100	1	-3.19

Seq1	hsa-miR-99a	1	-3.19
Seq1	hsa-miR-99b	1	-3.19
Seq1	hsa-miR-577	2	-3.18
Seq1	hsa-miR-10b	3	-3.17
Seq1	hsa-miR-603	3	-3.13
Seq1	hsa-miR-542-5p	2	-3.06
Seq1	hsa-miR-454	1	-2.94
Seq1	hsa-miR-628-5p	1	-2.94
Seq1	hsa-miR-613	6	-2.93
Seq1	hsa-miR-935	1	-2.92
Seq1	hsa-miR-562	1	-2.91
Seq1	hsa-miR-498	4	-2.89
Seq1	hsa-miR-1283	3	-2.85
Seq1	hsa-miR-758	5	-2.81
Seq1	hsa-miR-553	4	-2.78
Seq1	hsa-miR-639	1	-2.76
Seq1	hsa-miR-1185	4	-2.73
Seq1	hsa-miR-22	1	-2.72
Seq1	hsa-miR-1324	3	-2.62
Seq1	hsa-miR-485-3p	3	-2.57
Seq1	hsa-miR-570	2	-2.54
Seq1	hsa-miR-325	1	-2.53
Seq1	hsa-miR-633	1	-2.43
Seq1	hsa-miR-29c	2	-2.4
Seq1	hsa-miR-376c	5	-2.4
Seq1	hsa-miR-615-3p	1	-2.19
Seq1	hsa-miR-29a	2	-2.15
Seq1	hsa-miR-155	3	-2.14
Seq1	hsa-miR-548a-5p	6	-2.02
Seq1	hsa-miR-548d-5p	6	-2.02
Seq1	hsa-miR-548c-5p	6	-1.98
Seq1	hsa-miR-518b	5	-1.91
Seq1	hsa-miR-513b	3	-1.77
Seq1	hsa-miR-383	1	-1.53
Seq1	hsa-miR-767-5p	2	-1.35
Seq1	hsa-miR-885-5p	2	-1.29
Seq1	hsa-miR-890	1	-1.12
Seq1	hsa-miR-411	6	-1.11
Seq1	hsa-miR-548h	6	-1.06
Seq1	hsa-miR-559	5	-1.01
Seq1	hsa-miR-1278	3	-1
Seq1	hsa-miR-337-3p	2	-0.99
Seq1	hsa-miR-300	1	-0.94
Seq1	hsa-miR-508-3p	1	-0.91
Seq1	hsa-miR-548a-3p	3	-0.85
Seq1	hsa-miR-320b	3	-0.72
Seq1	hsa-miR-320a	3	-0.68

Seq1	hsa-miR-208b	3	-0.51
Seq1	hsa-miR-611	1	-0.5
Seq1	hsa-miR-1284	1	-0.4
Seq1	hsa-miR-598	2	-0.034
Seq1	hsa-miR-216a	2	0.08
Seq1	hsa-miR-586	2	0.09
Seq1	hsa-miR-369-3p	2	0.21
Seq1	hsa-miR-597	1	1
Seq1	hsa-miR-301a	1	1.05
Seq1	hsa-miR-384	2	1.34
Seq1	hsa-miR-548m	3	1.34
Seq1	hsa-miR-653	2	1.44
Seq1	hsa-miR-124	2	1.79
Seq1	hsa-miR-320c	3	2.01
Seq1	hsa-miR-494	1	2.2
Seq1	hsa-miR-130b	1	2.3
Seq1	hsa-miR-130a	1	2.65
Seq1	hsa-miR-875-5p	2	2.74
Seq1	hsa-miR-21	1	2.78
Seq1	hsa-miR-320d	3	2.82
Seq1	hsa-miR-142-5p	3	3.05
Seq1	hsa-miR-888	1	3.29
Seq1	hsa-miR-410	2	3.51
Seq1	hsa-miR-1277	1	4.24
Seq1	hsa-miR-340	1	5.24
Seq1	hsa-miR-301b	1	5.25
Seq1	hsa-miR-429	2	6.4
Seq1	hsa-miR-380	1	6.66
Seq1	hsa-miR-487b	1	6.93
